# Glutamine Reduces the Apoptosis of H9C2 Cells Treated with High-Glucose and Reperfusion through an Oxidation-Related Mechanism

**DOI:** 10.1371/journal.pone.0132402

**Published:** 2015-07-06

**Authors:** Kai Li, Yong-Chun Cui, Hong Zhang, Xiao-Peng Liu, Dong Zhang, Ai-Li Wu, Jian-Jun Li, Yue Tang

**Affiliations:** 1 Animal Experimental Center, Beijing Key Laboratory of Preclinical Research and Evaluation for Cardiovascular Implant Materials, State key Laboratory of Cardiovascular Disease, Fu Wai Hospital, National Center for Cardiovascular Disease, Chinese Academy of Medical Sciences and Peking Union Medical College, Beijing, China; 2 Division of Dyslipidemia, State Key Laboratory of Cardiovascular Disease, Fu Wai Hospital, National Center for Cardiovascular Disease, Chinese Academy of Medical Sciences and Peking Union Medical College, Beijing, China; Medical University of Graz, AUSTRIA

## Abstract

Mitochondrial overproduction of reactive oxygen species (ROS) in diabetic hearts during ischemia/reperfusion injury and the anti-oxidative role of glutamine have been demonstrated. However, in diabetes mellitus the role of glutamine in cardiomyocytes during ischemia/reperfusion injury has not been explored. To examine the effects of glutamine and potential mechanisms, in the present study, rat cardiomyoblast H9C2 cells were exposed to high glucose (33 mM) and hypoxia-reoxygenation. Cell viability, apoptosis, intracellular glutamine, and mitochondrial and intracellular glutathione were determined. Moreover, ROS formation, complex I activity, membrane potential and adenosine triphosphate (ATP) content were also investigated. The levels of S-glutathionylated complex I and mitochondrial apoptosis-related proteins, including cytochrome c and caspase-3, were analyzed by western blot. Data indicated that high glucose and hypoxia-reoxygenation were associated with a dramatic decline of intercellular glutamine and increase in apoptosis. Glutamine supplementation correlated with a reduction in apoptosis and increase of glutathione and glutathione reduced/oxidized ratio in both cytoplasm and mitochondria, but a reduction of intracellular ROS. Glutamine supplementation was also associated with less S-glutathionylation and increased the activity of complex I, leading to less mitochondrial ROS formation. Furthermore, glutamine supplementation prevented from mitochondrial dysfunction presented as mitochondrial membrane potential and ATP levels and attenuated cytochrome c release into the cytosol and caspase-3 activation. We conclude that apoptosis induced by high glucose and hypoxia-reoxygenation was reduced by glutamine supplementation, via decreased oxidative stress and inactivation of the intrinsic apoptotic pathway.

## Introduction

Clinical studies have shown that patients with diabetes mellitus (DM) are at higher risk of cardiovascular events compared with individuals without DM [1,2]. Mechanisms underlying the vulnerability of DM patients to myocardial ischemia are complicated and not fully understood. Several studies have suggested that the overproduction of reactive oxygen species (ROS) by mitochondria may be a core mechanism involved in aggravated ischemic injury after reperfusion in diabetic hearts [3,4].

Glutathione (GSH), the principle non-enzymatic cellular antioxidant, is vital to the regulation of intracellular oxidative balance. A consistent decrease in mitochondrial GSH (mtGSH) may be related to numerous pathologies, including hypoxia/reperfusion injury [5,6] and diabetes-associated diseases [7,8]. Moreover, depletion of mtGSH may cause mitochondrial protein S-glutathionylation, a post-transcriptional modification [9]. In addition, mitochondria complex I, the first and largest component within the electron transport chain, is sensitive to S-glutathionylation. S-glutathionylation of complex I can lead to activity loss and superoxide anion overproduction, resulting in ROS increase and cell apoptosis [10,11].

It has been demonstrated that glutamine (Gln), the precursor of glutathione, has the ability to decrease oxidative stress and protect the mesenterium [12] from ischemia/reperfusion (I/R) injury. Its protective role is associated with increased superoxide dismutase activity. Recently, several studies also demonstrated that Gln could alleviate I/R injury in the liver [13] and heart [14] by increasing the reduced GSH/oxidized GSH (GSSG) ratio. However, it is unknown whether Gln has the same protective role in diabetic hearts with I/R injury.

The present study investigated the role of Gln on I/R injury in diabetic hearts. Using rat cardiomyoblast H9C2 cells treated with high glucose and hypoxia/reoxygenation (H/R), we explored the effect of Gln on I/R diabetic hearts, and the potential mechanism of this effect.

## Materials and Methods

### Cell culture and treatment

Embryonic rat heart-derived H9C2 cells obtained from the Cell Resource Center of Peking Union Medical College were cultured in Dulbecco’s modified Eagle’s medium (DMEM, Cat. no. 11054–020, Invitrogen, Grand Island, NY, USA) supplemented with Gln (4 mM, Cat. no. 21051–024, Invitrogen), glucose (5.5 mM, Cat. no. 15023–021, Invitrogen), 10% fetal bovine serum (FBS; Invitrogen) and 1% penicillin–streptomycin (Invitrogen) at 37°C in a humidified atmosphere containing 5% CO_2_ and 95% air. Cells were subcultured at a 1:3 ratio every 3 to 4 days in 75 cm^2^ tissue culture flasks. Cell cultures between passages 3 to 5 were used for each experiment.

For the treatment procedure, H9C2 cells were cultured in low glucose (5.5 mM) with mannitol (untreated control) or high glucose (33 mM) in DMEM for 72 hours with different concentrations of Gln (0.5, 1, 2, 4, 8, 16, or 32 mM). To simulate hypoxia, the cell culture medium was replaced with Tyrode’s solution containing the following without glucose: 130 mM NaCl, 5 mM KCl, 10 mM HEPES, 1 mM MgCl_2_, and 1.8 mM CaCl_2_ at pH 7.4/37°C [15]. H9C2 cells were exposed to this solution in a controlled hypoxic chamber for 3 hours. Reoxygenation was conducted in a normoxic incubator at 37°C by replacing the ischemia medium for 3 hours with DMEM supplemented with 10% FBS containing the original respective Gln concentrations.

### Measurement of cell viability

Cell viability was measured using an MTT assay (M5655, Sigma-Aldrich, Saint Louis, MO, USA), based on the MTT conversion into formazan crystals using mitochondrial dehydrogenases. Briefly, H9C2 cells were plated at a density of 2 × 10^3^ cells/well in 96-well plates. After the treatment, the culture medium was replaced with 200 μL of MTT solution, that is, 5 mg/mL stock solution in phosphate buffered saline (PBS), diluted with culture medium to the final concentration of 0.5 mg/mL. After a 4-hour incubation at 37°C, this solution was removed and the formazan was solubilized in 150 μL of dimethyl sulfoxide. The absorbance was measured at 570 nm using an automated microplate reader (Tecan Infinite 200 pro microplate reader, Männedorf, Switzerland). Cell viabilities were calculated by comparing results to control cells considered 100% viable.

### Measurement of intracellular glutamine

Intracellular Gln level was assessed using a commercially available Gln measurement kit (A037, Nanjing Jiancheng Bioengineering Institute, Jiangsu Province, China) in accordance with the manufacturer’s instructions and expressed as percentage relative to the control value.

### TUNEL assay

The TUNEL (TdT-mediated dUTP nick-end labeling) assay was performed using a commercially available *in situ* apoptosis detection kit (No.11684795910, Roche Molecular Biochemicals, Mannheim, Germany) in accordance with the manufacturer’s protocol, as described previously [16]. H9C2 cells grown on 6-mm plates were fixed with 4% paraformaldehyde solution for 30 min at room temperature. After a rinse with PBS, cells were treated with permeation solution (0.1% Triton X-100 in 0.1% sodium citrate) for 2 min at 4°C. After washing with PBS, samples were incubated with TUNEL reagent containing terminal deoxynucleotidyltransferase and fluorescent isothiocyanate-dUTP. The cells were also stained with 1 μg/mL DAPI (4', 6-diamidino-2-phenylindole) for 30 min to detect the cell nuclei (indicated in blue). Using an excitation wavelength in the range of 450–500 nm and detection in the range of 515–565 nm (green), the number of TUNEL-positive cardiac cells and apoptotic bodies were determined. The percentage of apoptotic cells were calculated by dividing the number of TUNEL-positive cells by the total number of cells visualized in the same field. Three digitized images of similar total cell numbers were selected from each cover slip for counting and averaging and were considered as one independent experiment. Three independent experiments were then averaged and statistically analyzed.

### Measurement of ROS release

Intracellular ROS were measured using 2’, 7’-dichlorofluorescein (DCF) diacetate (DA) (D6883, Sigma-Aldrich, Saint Louis, MO, USA) and dihydroethidium (D11347, Molecular Probes, Eugene, OR, USA) in accordance with the manufacturer’s protocol, as described previously [17,18,19,20]. The fluorescence intensity of each group was measured by flow cytometry (BD Accuri C6) and expressed as a percentage relative to the control value.

### Preparation of cytosolic and mitochondrial fractions

Preparation of cytosolic and mitochondrial fractions was achieved using a commercially available cytosolic/mitochondrial fractionation kit in accordance with the manufacturer's protocol (C3601, BiYunTian, Institute of Biotech, Shanghai, China) as previously described [21]. Briefly, 3 × 10^7^ cells were washed thrice with ice-chilled PBS at 600×*g*. Cell pellets were resuspended in 2 mL of extraction buffer and incubated at 4°C for 20 min, followed by homogenization. The homogenate was centrifuged at 600×*g* for 10 min at 4°C. The supernatant was additionally centrifuged at 11,000×*g* for 10 min (fraction enriched with intact mitochondria). The supernatant from the former centrifugation was used as the cytosolic fraction and the final pellet was a more purified mitochondrial fraction. The mitochondrial and cytosolic fractions were essentially free of other compartmental contaminants (S1 Fig), as previously reported [22,23].

### Evaluation of GSH and, GSSG in the cytosolic and mitochondrial fractions

Cytosolic and mitochondrial GSH and GSSG levels were detected in accordance with the directions in the Total Glutathione Assay Kit (S0053, BiYunTian, Institute of Biotech, Shanghai, China) as previously described [24]. The absorbance values were measured at 412 nm. Cytosolic and mitochondrial GSH, GSSG levels and GSH/GSSG ratio were normalized to that of the control groups.

### Measurement of mitochondrial membrane potential

The mitochondrial membrane potential was assessed using the fluorescent indicator 5,5’,6,6’- tetrachloro- 1,1’,3,3’-tetraethyl benzimidazolylcarbcyanine iodide (JC-1) in accordance with the manufacturer’s protocol (No.70014, Biotium, Hayward, CA, USA). To measure the quantitative change in mitochondrial potential, we assessed JC-1 with a fluorescence plate reader. Briefly, 2 × 10^3^ cells in 200 μL culture medium/well were seeded in a black 96-well plate (Nunc, Denmark) and after the treatment, JC-1 (2.5 μg/mL) was added for 30 min at 37°C in the dark. Cells were washed twice with PBS to remove unbound dye. The mitochondrial membrane potential was expressed as a ratio of fluorescent emission (580/520 nm) normalized to the control fluorescent baseline. Cells were also viewed and photographed under a Leica confocal microscope.

### Determination of intracellular ATP level

Intracellular ATP was determined using an ATP assay kit (S0026, BiYunTian, Institute of Biotech, Shanghai, China) based on a luciferin/luciferase assay and normalized for protein content as described earlier [25]. Light emission was measured with a Luminescence Analyzer. Raw data were collected as relative light units integrated over 20 s for samples and converted to ATP concentrations with the aid of a standard calibration curve obtained using ATP standards. The ATP concentrations were calculated from standard curve data and expressed as nmol per mg protein.

### Enzymatic analysis of mitochondrial complex I activity

The activity of mitochondrial complex I was determined immediately after isolation of the mitochondrial fraction. Complex I activity was analyzed using an immunocapture complex I enzyme activity assay (MS141, MitoSciences, Cambridge, UK) in accordance with the manufacturer's recommendations.

### Measurement of mitochondrial ROS formation

Mitochondrion-derived ROS in H9C2 cells were determined by an ELISA method with MitoSOX red reagent (M36008, Molecular Probes, Eugene, OR, USA), in accordance with the manufacturer’s protocol. The intensity of fluorescence was determined semi-quantitatively in conjunction with a Leica confocal microscope.

### Immunoblot

Immunoblot was performed as previously described [15]. Proteins were prepared from the cytosolic and mitochondrial samples or whole-cell lysis of the H9C2 cells. Protein concentrations were measured using a bicinchoninic acid assay (Biotech). Equal amounts of protein (30 μg/lane) were resolved by electrophoresis for western blot analysis. The proteins were transferred to polyvinylidene difluoride membranes (Millipore) using a semi-dry electroblot apparatus (Bio-Rad).

The membranes were blocked for 1 h at room temperature in 5% skim milk and incubated serially overnight at 4°C with the following primary antibodies: cytochrome c antibody (#4272; 1:1000), caspase-3 antibody (#9662; 1:1000), β-actin antibody (#12620; 1:1000) from Cell Signal Technology; and voltage-dependent anion selective channel protein 1 (VADC1) antibody (sc-390996; 1:100), NADH dehydrogenase (ubiquinone) flavoprotein 1 (NDUFV1) antibody (sc-133808; 1:200), and NADH dehydrogenase (ubiquinone) Fe-S protein 1 (NDUFS1) antibody (sc-99232; 1:200) from Santa Cruz Biotechnology. After washing, secondary antibody (sc-2030 and sc-2031, 1:2000 dilution, Santa Cruz Biotechnology) was added to the membrane for 1 h at room temperature.

The membranes were washed and processed for analysis using a Chemiluminescence Detection Kit (P90719, Millipore, Billerica, MA, USA) in accordance with the manufacturer's instructions. The target signals were normalized to the β-actin or VADC1 signal and analyzed semi-quantitatively with a FluorChem M System (ProteinSimple, San Jose, CA, USA).

### Detection of protein S-glutathionylation with immunoprecipitation (IP)

Mitochondrial samples were prepared and ultrasonicated. The mitochondrial protein concentration was measured using a bicinchoninic acid assay (Biotech). Immunoprecipitation was conducted in accordance with the manufacturer's protocol (Protein A/G PLUS-Agarose Immunoprecipitation Reagent: sc-2003, Santa Cruz, CA, USA). Briefly, equal amounts from mitochondrial protein (100 μg/each group) were transferred to a 1.5 mL microcentrifuge tube. One microgram of NDUFV1 or NDUFS1 antibody was added and incubated for 1 h at 4° C. Nonspecific mouse IgG was used as a negative control. Then, 20 μL of resuspended volume of Protein A/G PLUS-Agarose was added and incubated at 4° C on a rotating device overnight.

Immunoprecipitates were collected by centrifugation at 1000×*g* for 5 min at 4° C. The radioactive supernatant was carefully aspirated and discarded. The pellet was washed 4 times with 1.0 mL RIPA buffer, and aspirated after the final wash; The supernatant was discarded and the pellet resuspended in 40 μL of 1× electrophoresis sample buffer. Boiled samples were washed for 3 min and analyzed 20 μL aliquots by SDS-PAGE, immunoblotted with anti-GSH monoclonal antibody (Virogen, Watertown, MA, USA, 1:1000 dilution) under non-reducing conditions, and visualized using a Chemiluminescence Detection Kit (P90719, Millipore corporation, Billerica, MA, USA). As a control, mitochondrial protein (20 μg) was also immunoblotted with NDUFV1 or NDUFS1 antibody.

### Statistical analysis

All values are presented as mean ± standard deviation. Statistical analysis was performed using Student’s *t*-test for two groups and one-way analysis of variance for multiple groups. *P* < 0.05 was considered statistically significant.

## Results

### Relation of Gln levels to H9C2 apoptosis

**Fig 1:**
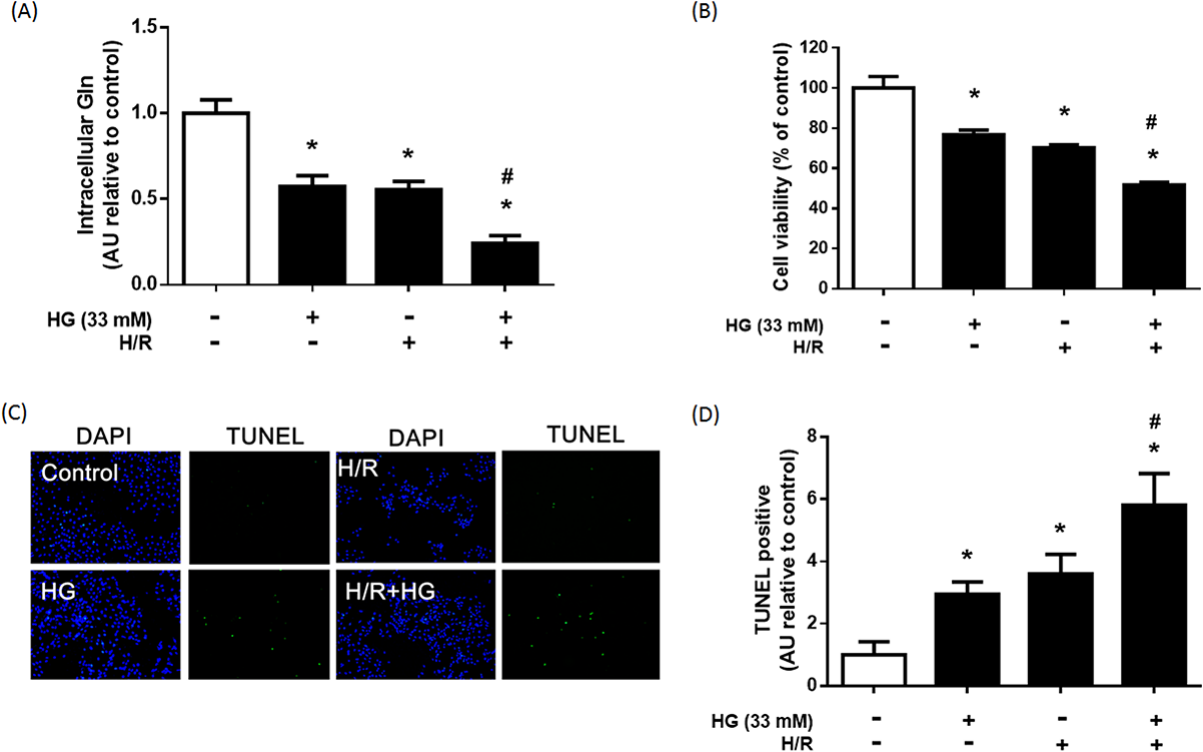
Effect of hyperglycemia (HG) and hypoxia/reperfusion (H/R) on intracellular Gln, cell viability, and apoptosis. H9C2 cells were treated with HG (33 mM), H/R, or HG + HR. (A) Intracellular Gln level in each group. (B) Cell viability of each group was established using an MTT assay. (C) HG and H/R-induced cell apoptosis was examined by DAPI staining and TUNEL assay. Blue spots represent cell nuclei and green spots represent apoptotic bodies. (D) Bars represent the percentage of TUNEL-positive cells based on the total number of cells stained by DAPI. The results are expressed as mean ± S.D. of 3 independent experiments. * *p* < 0.05 compare with the control group; # *p* < 0.05 compare with the HG and H/R group; a.u. = arbitrary units.

We examined whether high glucose and H/R could decrease intracellular Gln levels. H9C2 cells were exposed to one of 4 treatments: 33 mM high glucose for 72 hours (HG); 3 hours hypoxia and 3 hours reoxygenation (H/R); 33 mM high glucose for 72 hours and then 3 hours hypoxia and 3 hours reoxygenation (HG+H/R); or not treated (control). Intracellular Gln levels were markedly lower in the HG or H/R groups than in the control group (*P* < 0.05; Fig 1A). Cells in HG+H/R group had the lowest intracellular Gln levels (*P* < 0.05).

MTT assay was performed to evaluate cell viability. The cell viability of HG or H/R groups was significantly lower than that of the control (*P* < 0.05; Fig 1B). Exposure to HG+H/R was associated with greater loss in cell viability compared with that of the HG and H/R groups (*P* < 0.05).

Cell viability was closely related to cell apoptosis. Cell apoptosis was evaluated with a TUNEL assay (Fig 1C and 1D). The number of apoptotic bodies in the HG+H/R group was markedly higher than in the HG or H/R groups (*P* < 0.05). These results revealed that high glucose and H/R can decrease intracellular Gln levels and induce H9C2 apoptosis.

**Fig 2:**
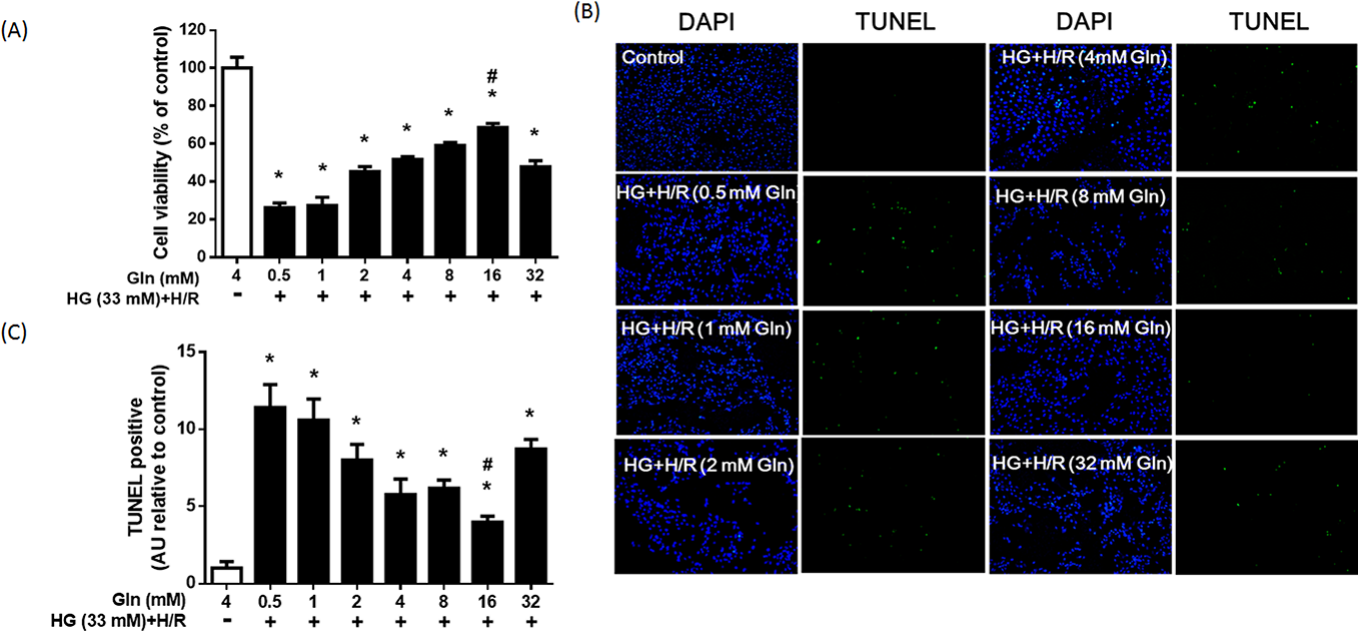
Effect of Gln on cell viability and apoptosis. H9C2 cells were exposed to HG + H/R with different concentrations of Gln (0.5, 1, 2, 4, 8, 16, and 32 mM). (A) Cell viability of each group was determined by MTT assay. (B) Results of the TUNEL assay and DAPI staining of each group. Green spots represented apoptotic bodies and blue spots represent cell nuclei. (C) Bars represent the percentage of TUNEL-positive cells based on the total number of cells stained by DAPI. * *P* < 0.05 compare with the control group, # *P* < 0.05 compare with the 1 mM and 4 mM Gln groups. Data are expressed as mean ± S.D. of 3 independent experiments; a.u. = arbitrary units.

To observe the effect of Gln on cell viability or apoptosis, H9C2 cells were exposed to high glucose and H/R, with various concentrations of Gln (0.5–32 mM). The MTT assay showed a concentration-dependent association between Gln treatment and cell viability (Fig 2A). In accord with the MTT results, the TUNEL assay also revealed a Gln concentration-dependent decrease in the number of TUNEL-positive nuclei in the high glucose and H/R treated cells, indicating that Gln could inhibit cell apoptosis (Fig 2B and 2C). The least number of apoptotic cells was found in the group treated with 16 mM Gln. More apoptotic cells were found in the group treated with 32 mM Gln than in those treated with Gln concentrations of 8–16 mM. The results suggested that apoptosis induced by high glucose and H/R could be reduced, at least partly, by Gln supplementation. Based on these results, in subsequent studies, 4 experimental treatments were used to elucidate the potential protective mechanism of Gln: HG+HR with 1, 4, or 16 mM Gln, and the control.

### Gln supplementation associated with less ROS and increased cytoplasmic GSH and GSH/GSSG

**Fig 3:**
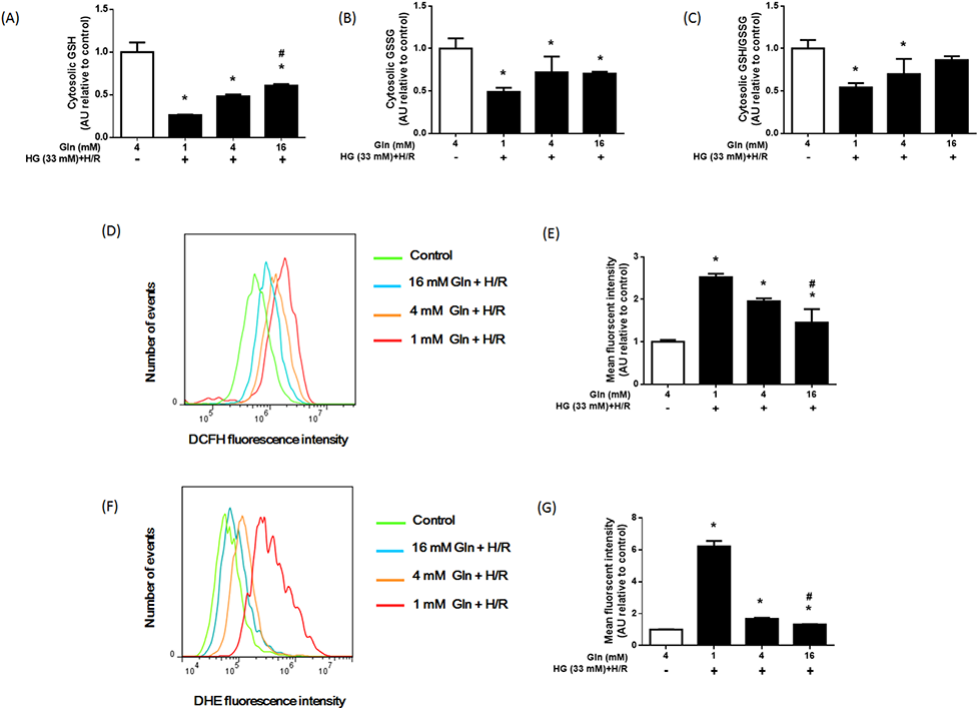
Effect of Gln on cytosolic GSH, GSSG and intracellular ROS. H9C2 cells were exposed to HG+H/R with different concentrations of Gln (1, 4, or 16 mM). (A) Cytosolic GSH, (B) GSSG, and (C) the GSH/GSSG ratio in each group were detected. (D) Representative images of DCFH flow cytometry of each group. (E) Mean DCFH fluorescence intensities are presented as percentage relative to the control value. (F) Representative images of DHE flow cytometry of each group. (G) Mean DHE fluorescence intensities are presented as percentage relative to the control value. These data are expressed as the mean ± S.D. of 3 independent experiments. * *P* < 0.05 compare with the control group, # *P* < 0.05 compare with the 1 mM Gln and 4 mM Gln groups, a.u. = arbitrary units.

Gln is a precursor amino acid of glutathione, which can improve cellular tolerance to oxidative stress. Furthermore, *de novo* GSH synthesis occurs exclusively in the cytosolic compartment [26]. To detect whether apparent anti-apoptotic effects of Gln were further associated with GSH-GSSG balance, cytoplasmic GSH, GSSG, and the GSH/GSSG ratio were measured (Fig 3A, 3B and 3C). Cytoplasmic GSH, GSSG, and GSH/GSSG levels were markedly lower in HG+H/R-treated H9C2 cells than in the control groups (*P* < 0.05). This effect was reversed by 16 mM Gln although the lower doses of Gln had no such effect.

To observe the effect of Gln on intracellular ROS generation, DCFH-DA and dihydroethidium assays were performed (Fig 3D–3G). The levels of intracellular ROS were significantly higher in the HG and H/R-treated H9C2 cells relative to the non-treated control cells (*P* < 0.05). Intracellular ROS levels in the HG and H/R groups treated with Gln were negatively associated with Gln concentration.

Taken together, Gln supplementation appeared to promote the synthesis of GSH and suppress free radical up regulation in H9C2 cells grown under high glucose and H/R conditions.

### Effect of Gln on GSH, complex I activity, and ROS formation in mitochondria

**Fig 4:**
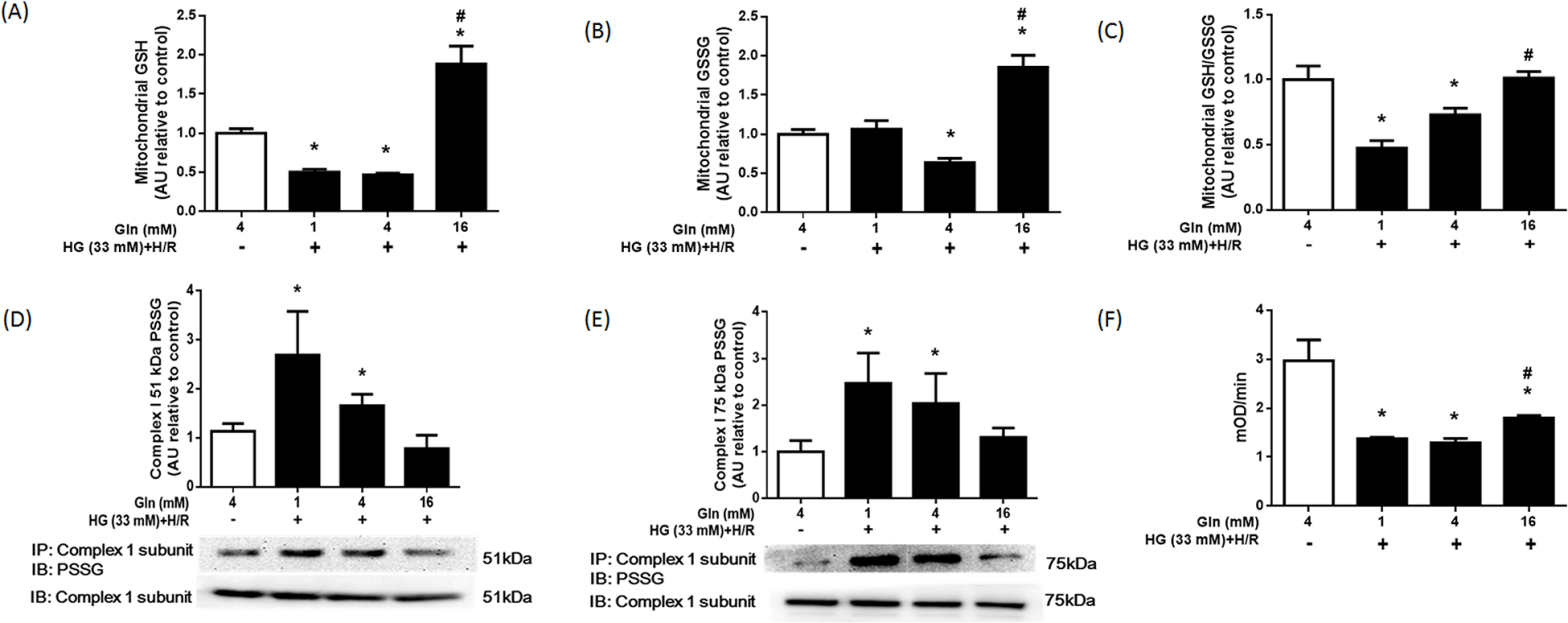
Effect of Gln on mitochondrial GSH, GSSG, S-glutathionylation, and complex I activity. H9C2 cells were treated as described in Fig 3. (A) Mitochondrial GSH, (B) GSSG, and (C) the GSH/GSSG ratio in each group were analyzed. (D and E) S-glutathionylated Complex I protein expression (51—and 75—kDa subunits) from each group were detected by immunoprecipitation and western blot analysis. The up panel shows the anti-complex I 75 and 51 kDa subunits positive-IP sample followed by identification by an anti-GSH antibody. As a control, mitochondrial protein (20 μg) was also immunoblotted with complex I 75 kDa and 51 kDa subunit antibody. Bar graphs summarize the protein band intensities of S- glutathionylated Complex I. (F) Complex I activity (200 μg mitochondrial protein) of each group are detected. * *P* < 0.05 compare with the control group, # *P* < 0.05 compare with the 1 mM Gln and 4 mM Gln groups. The data are expressed as the mean ±S.D. of 3 independent experiments, a.u. = arbitrary units.

As the primary antioxidant in mitochondria, GSH also has an important role in mitochondrial oxidative stress [27]. Here, we investigated the effect of Gln on mtGSH levels in cells exposed to high glucose and H/R (Fig 4A, 4B and 4C). The levels of mtGSH and mtGSH/GSSG in the HG+H/R groups treated with low-dose Gln (1 mM or 4 mM) were significantly lower than that of the control group (*P* < 0.05).

Interestingly, mtGSH levels in the HG+H/R cells treated with the 16 mM Gln group were significantly higher compared with HG+H/R cells treated with 1 mM or 4 mM Gln (*P* < 0.05), or even the control group (*P* < 0.05). The decrease in the mtGSH/GSSG ratio in the 1 mM and 4 mM Gln groups associated with exposure to HG and H/R was not observed in the 16 mM Gln group. These results indicated that Gln supplementation may have a role in reducing mitochondrial oxidative stress in cardiac cells exposed to high glucose and H/R.

A decrease in the mtGSH or mtGSH/GSSG ratio can lead to a change in mitochondrial redox status, which may induce the S-glutathionylation of complex I and cause activity loss, as well as increase ROS generation [28]. Therefore, we assessed the effect of Gln on complex I in H9C2 cells exposed to HG and H/R (Fig 4D and 4E). S-glutathionylation of the 51-kDa subunit of complex I in HG+H/R cells treated with 1 mM and 4 mM Gln was higher than that of the control group (*P* < 0.05). S-glutathionylation of the 51-kDa subunit of Complex I in the 16 mM Gln group was similar to that in control group (*P* > 0.05). Similar results were obtained from S-glutathionylation of the 75-KDa subunit. S-glutathionylation of the 75-KDa subunit of complex I was significantly higher in the 1 mM and 4 mM Gln groups (*P* < 0.05) than in the control group, but this was not true of the 16 mM Gln group (*P* > 0.05).

**Fig 5:**
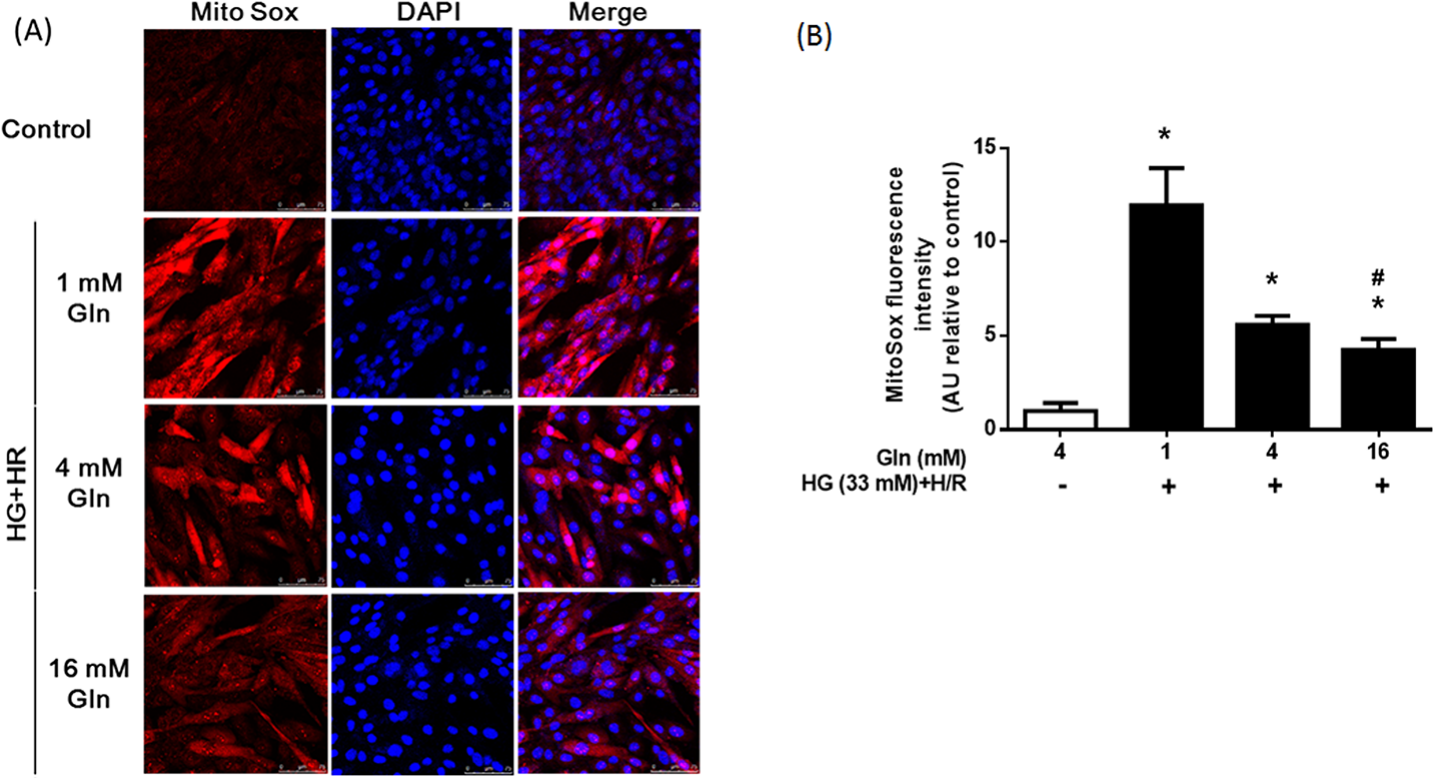
Effect of Gln on mitochondrial ROS formation. H9C2 cells were treated as described in Fig 3. (A) Representative imaging of Mito Sox confocal image of each group. (B) Mean Mito Sox fluorescence intensities are presented as percentage relative to the control value. The data are expressed as the means ±S.D. of 3 independent experiments. * *P* < 0.05 compared with the control group; # *P* < 0.05 compare with the 1 mM and 4 mM Gln groups. a.u. = arbitrary units.

We next examined complex I activity and mtROS production in the control and HG+H/R-treated groups. Treatment of the HG and H/R significantly decreased the activity of complex I (*P* < 0.05, Fig 4F). The complex I activity in the 1 mM, 4 mM, and 16 mM Gln groups was 44%, 46%, and 61% that of the control group, respectively. Mitochondria-derived ROS levels in the HG + H/R groups were significantly higher than that of the control group (*P* < 0.05, Fig 5A and 5B). However, the increase was not observed in the 16 mM Gln group (*P* < 0.05).

These results indicated that Gln supplementation largely prevented S-glutathionylation, activity loss of complex I, and decreased mitochondria-derived ROS formation in cardiac cells subjected to high glucose and H/R.

### Influence of Gln on mitochondrial transmembrane potential (ΔΨm) and ATP levels

**Fig 6:**
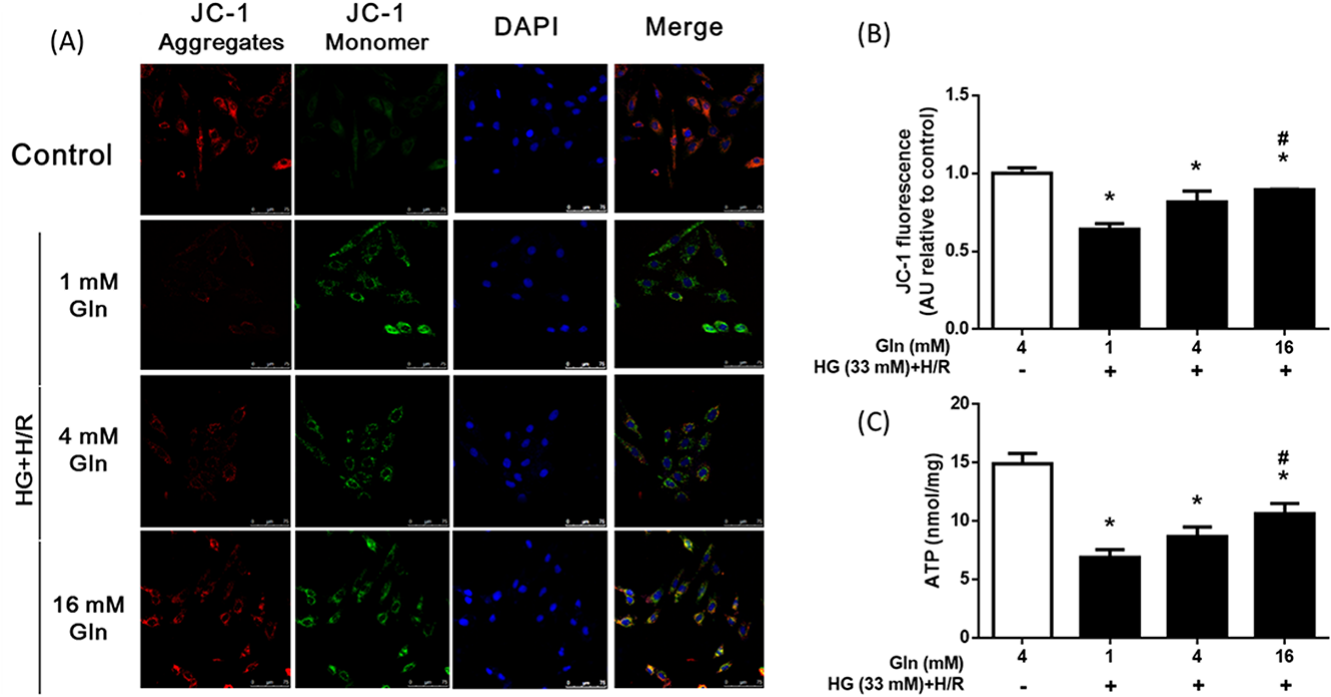
Effects of Gln on mitochondrial membrane potential stability and ATP level. H9C2 cells were treated as described in Fig 3. (A). Representative images of JC-1 confocal images of each group; the red image detected in the left panel represents JC-1 aggregates, and the green image represents JC-1 monomer; the blue image represents DAPI, right panel shows the merged image. (B) Mean ± S.D. of the changes in radiometric JC-1 fluorescence for each group as indicated. (C) Cellular ATP levels of each group were detected. * *P* < 0.05 compare with the control group; # *P* < 0.05 compare with the 1 mM Gln and 4 mM Gln groups. Data are expressed as the mean ±S.D. of 3 independent experiments. a.u. = arbitrary units.

Mitochondrial transmembrane potential (ΔΨm) is a marker of mitochondrial function, and dissipation of ΔΨm always indicates mitochondrial dysfunction [28]. In the present study, ΔΨm of the control group was normal, evident from the significant amount of JC-1 aggregates (red fluorescence; Fig 6). On the other hand, HG + H/R treatment depolarized ΔΨm (decreasing the red/green fluorescence ratio) relative to the control group (*P* < 0.05). The ratio of red-to-green fluorescence was significantly higher in the 16 mM Gln group than in the 1 mM and 4 mM groups (*P* < 0.05).

ATP generation is also an essential marker for mitochondrial function [28]. In the present study, ATP levels of HG+H/R group were significantly lower than that of the control group, which was alleviated more by 16 mM Gln treatment (Fig 6C; *P* < 0.05). These results suggested that Gln supplementation largely preserved mitochondrial function under high glucose and H/R conditions in cardiac cells.

### Influence of Gln in mitochondrial apoptosis-related proteins

Much evidence indicates that mitochondrial dysfunction results in the release of cytochrome c into the cytosol, which is considered a hallmark of apoptosis in the mitochondrial pathway [6]. Caspase-3 is considered an executioner caspase. Its cleavage or activity is another marker of apoptosis [29]. Therefore, we examined the levels of protein cytochrome c in isolated mitochondrial and cytosolic fractions, and cleaved caspase-3 and pro caspase-3 protein in control and HG+H/R groups.

**Fig 7:**
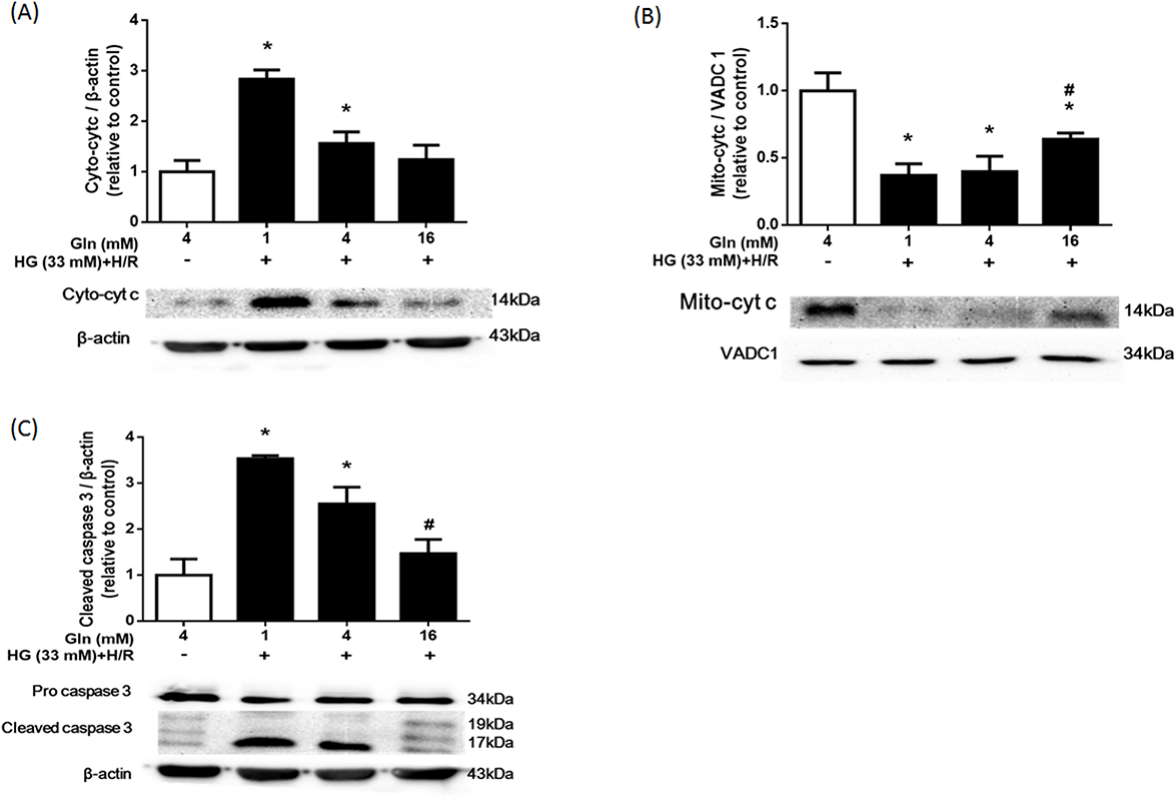
Influence of Gln on mitochondrial apoptosis-related protein. H9C2 cells were treated as described in Fig 3. The expression of cytosolic (A) and mitochondrial (B) cytochrome c (cyto-c) as well as cleaved caspase-3 and pro caspase-3 (C) in each group were measured by western blot. Beta-actin and VADC1 were used as internal protein loading controls. Bar graphs summarize the protein band intensities of cyto-c and cleaved caspase 3. * *P* < 0.05 compare with the control group; # *P* <0.05 compare with the 1 mM and 4 mM Gln groups.

Western blot analysis revealed that in the 1 mM and 4 mM Gln groups exposed to high glucose and H/R, the levels of cytochrome c in mitochondrial fractions was higher, and the levels of cytochrome c in cytosolic fractions was lower, compared with the control (Fig 7A and 7B). No significant difference in these fractions was found in HG+H/R cells exposed to 16 mM Gln. Furthermore, in the 1 mM and 4 mM Gln groups, the levels of cleaved caspase 3 at 17-and 19-kDa were significantly higher than that of the control cells, but these higher levels were not observed in the cells treated with 16 mM Gln (Fig 7C).

These results indicated that 16 mM Gln supplementation can prevent release of cytochrome c from the mitochondria and protect cardiac cells from high glucose and H/R-induced cell apoptosis through the mitochondrial pathway.

## Discussion

**Fig 8:**
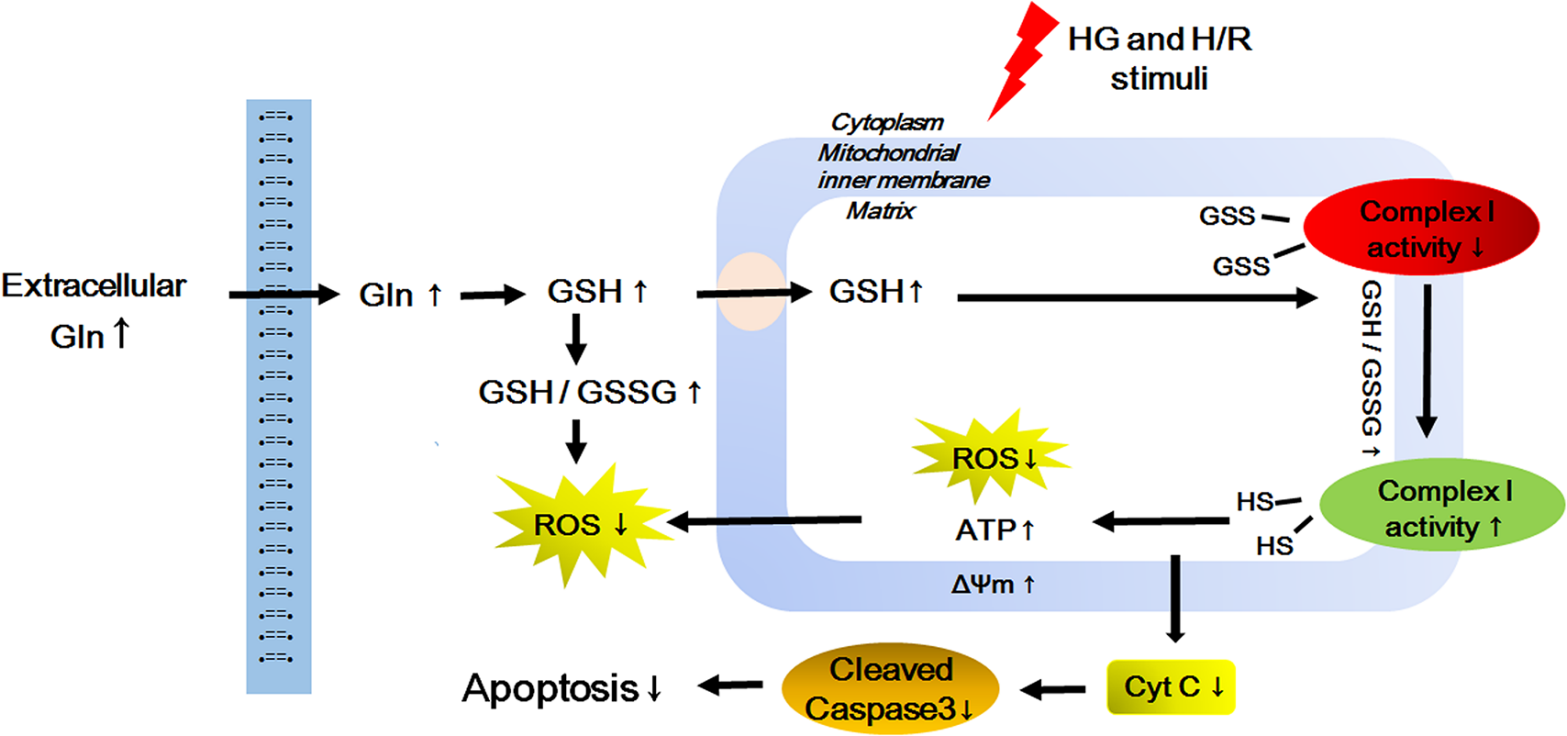
Possible mechanism of the protective effect of Gln on HG and H/R-treated H9C2 cells. Abbreviations: HG = high glucose; H/R = hypoxia-reoxygenation; GSH = glutathione; GSSG = oxidized glutathione; ROS = reactive oxygen species; ATP = adenosine triphosphate.

In the present study, we found for the first time that Gln has an anti-apoptotic effect on cardiomyocytes during H/R under high glucose conditions. This protective role is likely by way of decreasing oxidative stress, and inactivation of the intrinsic apoptotic pathway (Fig 8). Therefore, our study may hint at a novel therapy for diabetic patients suffering from I/R injury.

Gln is the most abundant amino acid in mammalian cells and a multifunctional amino acid, with important roles in many metabolic pathways [30]. Although Gln is classified as a non-essential amino acid, evidence suggests that Gln is markedly depleted in the blood during stressful situations such as surgery and exhaustive exercise [30]. For these reasons, Gln is regarded as a conditional-essential amino acid under pathological conditions. In this study, we found that treatment of H9C2 cells with high glucose and H/R led to a significant drop in the levels of intracellular Gln (Fig 1), and that Gln supplementation preserved cell viability and lowered apoptosis rates (Fig 2). These findings strongly indicate that Gln protects against cell damage due to high glucose and H/R.

The overproduction of ROS is associated with a wide range of pathological conditions, including I/R injury, degenerative diseases, diabetes, and aging [31]. At low concentrations, ROS are essential signaling molecules, while excessive ROS can be detrimental. ROS can cause oxidation of membrane phospholipids, proteins and DNA, and can induce cell death [32]. To counteract redox imbalance, cells have defense mechanisms to fight against oxidation.

GSH is the main non-enzymatic antioxidant in the cytoplasm and mitochondria [29]. As an important substrate for GSH synthesis, Gln may have a role in regulation of the intracellular oxidative balance. In this study, we found that Gln supplementation increased GSH and GSH/GSSG, both in the cytoplasm and mitochondria and decreased intercellular ROS (Figs 3 and 4). These results indicated that, Gln (via GSH) could act as a ROS scavenger, to decrease intracellular oxidative stress in diabetic cardiomyocytes with I/R injury. These results are also consistent with previous studies [14] reporting that increased GSH/GSSG is the main protective effect of Gln against cardiac I/R injury.

Complex I is the first and largest component within the electron transport chain [33]. In addition to electron transfer and ATP production, complex I is also a major source of superoxide anion radical production [34], especially when complex I is damaged or its activity is decreased. Overproduction of superoxide anions and oxidants derived from it can change the redox status in mitochondria and cells, and leads to serious consequences [6]. Complex I is also the main host of reactive protein thiols in mitochondria. During oxidative stress (high GSSG or low GSH/GSSG), S-glutathionylation (the reversible formation of a mixed disulfide between protein thiols and GSSG) of complex I will occur.

Previous studies reported that the redox status of these thiols can influence complex I activity and electron transport chain function. Passarelli et al. [35] reported that complex I is highly susceptible to S-glutathionylation under conditions of oxidative stress, and complex I activity is strongly inhibited by S-glutathionylation in cardiac mitochondria. Similar results were demonstrated by Wu et al. [36] in the mitochondria of lens epithelial cells. Similarly, Beer et al. [11] showed that deglutathionylation of complex I by glutaredoxin 2, a mitochondrial isozyme of glutaredoxin, could re-activate or protect against complex I activity loss. Since GSH is an important antioxidant in mitochondria [29], increases in mtGSH levels may be a way to attenuate oxidative stress in mitochondria. In this study, we found that high glucose and H/R decreased mtGSH levels, while Gln enrichment can dramatically upregulate mtGSH and thereby increase the GSH/GSSG ratio, bringing the redox status back to normal. We also found that Gln enrichment inhibited S-glutathionylation of the 75- and 51- kDa subunits of complex I, and afterward increased complex I activity, decreased mitochondrial ROS formation. Gln deprivation had the reverse effect. These results indicated that Gln may ameliorate oxidative stress in mitochondria and suppress mitochondria ROS overproduction during I/R injury in diabetic hearts.

The function of mitochondria is closely linked to maintenance of redox. Mitochondria are susceptible to the damaging effect of ROS, and are the main generator of ROS. The accumulation of ROS in mitochondria always results in mitochondrial dysfunction, and correlates with dissipation of the ΔΨm, cessation of ATP generation, and mtDNA fragmentation, leading to cell death. In the present study, in cells treated with 16 mM Gln, decline in ΔΨm loss and ATP levels were alleviated (Fig 6). This supports the notion that, at least in part, the protective effect of Gln is due to the prevention of mitochondrial dysfunction. These results are consistent with previous studies showing the ability of Gln to enhance the mitochondrial activity of cells exposed to hyperoxia [37] or endotoxin [38]. Mitochondrial cytochrome c release from mitochondria and downstream caspase activation are both important in regulating apoptosis of cardiomyocytes [39]. Our study showed that supplemental Gln prevented the release of cytochrome c from mitochondria and attenuated caspase 3 activation, thereby reducing the apoptosis that is induced by high glucose and H/R (Fig 7). In summary, our data suggests that Gln has an anti-apoptotic effect, inhibiting the intrinsic signaling pathways associated with apoptosis.

Oxidative stress links multiple risk factors to disease, including cardiovascular disease. Many nutrients decrease oxidative stress [40], including the antioxidants: vitamin C, vitamin E, and folic acid. Drugs that possess indirect antioxidant properties, such as statins, angiotensin-converting enzyme inhibitors, or AT1-receptor blockers, have been proposed to restore redox status. In diabetes, it is becoming clear that a more comprehensive approach should aim at not only scavenging reactive radicals, but also preventing the generation of these reactive species [41]. In the present study, we found that Gln can act as a ROS scavenger by increasing GSH levels as well as by preventing the overproduction of mitochondrial ROS. Our results suggest that application of Gln may be a promising tool to reduce I/R injury in DM patients.

The present study has several limitations. Firstly, it should be emphasized that these experiments were performed with H9C2 cells. Thus, our observations cannot be fully extrapolated to the *in vivo* environment. Secondly, GSH cannot be synthesized in mitochondria *de novo* [6]. Therefore, mitochondrial GSH arises from cytosol GSH specific carriers [29], and the role of transporters of GSH was not determined in our study. Thirdly, we focused only on the intrinsic pathway of apoptosis, but extrinsic pathways may also have a role. Fourthly, fluorescence-based assays (e.g., flow cytometry, confocal microscopy) are not precise methods to assess intracellular ROS using dihydroethidium or MitoSOX assay [42]. As MitoSOX-derived fluorescence can be affected by many factors [42], (e.g., DNA binding, apoptosis, and necrosis), we may have overestimated the effects of low concentration Gln on absolute mitochondrial ROS production. Finally, many other signaling pathways have been implicated in the protective function of Gln, including increased O-GlcNAc (β-linked N-acetylglucosamine) [43] and heat shock protein (HSP)72 levels [14]. Further studies are required in the future.

## Conclusions

Our current study indicated that the protective mechanism of Gln in H9C2 cells that had been subjected to oxidative stress and apoptosis is likely associated with increased the GSH levels in both cytoplasm and mitochondria. The increase in GSH thereby decreases mitochondrial oxidative stress and inhibits complex I glutathionylation, which finally preserves the biological function of mitochondria. Accordingly, Gln supplementation may be a novel strategy for cardioprotection in DM patients at risk for cardiac I/R injury.

## Supporting Information

S1 Fig Identity and purity of mitochondrial fraction. Isolated mitochondria, cytosol were analyzed to check for contamination. (A) Mitochondria were corroborated by transmission electron microscopy. (B) Mitochondrial fractions were labelled with MitoTracker green and analyzed under a fluorescence microscope. (C) Mitochondrial, nuclear, and cell debries were labelled with MitoTracker green and propidium iodide, then analyzed on a flow cytometer. Fluorescence intensity in arbitrary units. (D) Western blot of the same fractions with antibodies for characteristic proteins of mitochondria (VADC1), cytosol (GAPDH) and nuclei (Histone-H3).
(TIF)
