# Supplementary figures and images for: Glutamine Reduces the Apoptosis of H9C2 Cells Treated with High-Glucose and Reperfusion through an Oxidation-Related Mechanism
Source: PLoS One. 2015 Jul 6;10(7):e0132402. doi: 10.1371/journal.pone.0132402 (PMC4493145; doi:10.1371/journal.pone.0132402)

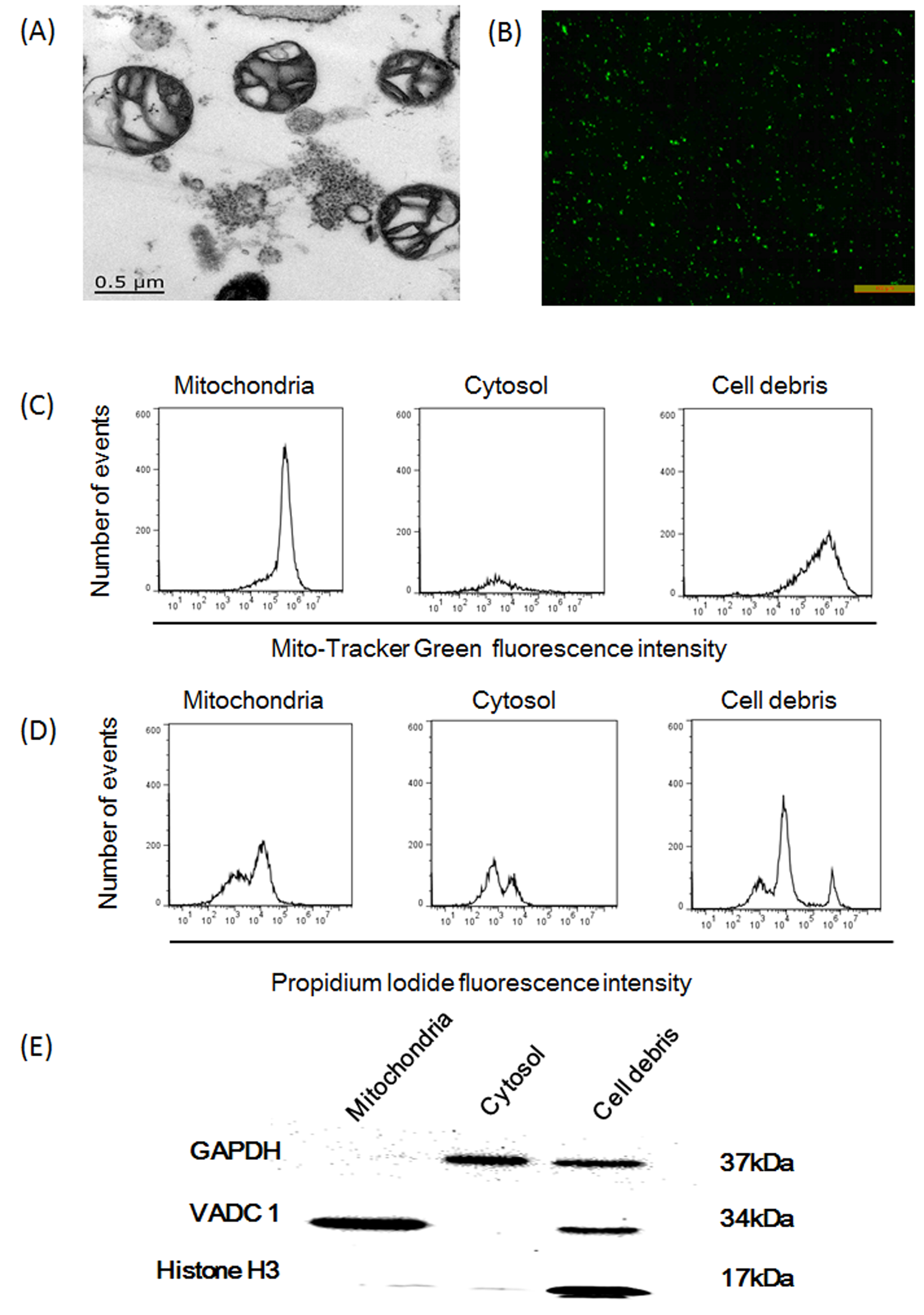

Supplement: S1 Fig — Isolated mitochondria, cytosol were analyzed to check for contamination. (A) Mitochondria were corroborated by transmission electron microscopy. (B) Mitochondrial fractions were labelled with MitoTracker green and analyzed under a fluorescence microscope. (C) Mitochondrial, nuclear, and cell debries were labelled with MitoTracker green and propidium iodide, then analyzed on a flow cytometer. Fluorescence intensity in arbitrary units. (D) Western blot of the same fractions with antibodies for characteristic proteins of mitochondria (VADC1), cytosol (GAPDH) and nuclei (Histone-H3). (TIF) [file pone.0132402.s001.tif]
